# Social Media Influence on Surgeon Selection Among Iranian Maxillofacial Patients: Cross-Sectional Survey Study

**DOI:** 10.2196/75899

**Published:** 2025-08-14

**Authors:** Mehdi Abrishami, Milad Bayat, Elham Sheykhi Some

**Affiliations:** 1 Department of Oral & Maxillofacial Surgery, Faculty of Dentistry, Isf. C, Islamic Azad University, Isfahan, Iran; 2 School of Dentistry, Islamic Azad University, Isfahan, Isfahan, Iran; 3 School of Health Management and Information Sciences, Iran University of Medical Sciences, No. 6, Shahid Yasami St, Vali‑e‑Asr Ave, Tehran, 19967 13883, Iran, 98 9037103664

**Keywords:** social media, patient selection, decision making, trust, oral surgery

## Abstract

**Background:**

Social media has reshaped health care decision-making; however, its influence on maxillofacial surgeon selection in non-Western contexts such as Iran remains underexplored. Understanding how patients balance digital platforms (eg, Google, Instagram) with traditional referral networks can inform trust dynamics and patient-centered care strategies.

**Objective:**

This study aimed to evaluate the impact of social media compared to personal recommendations on maxillofacial surgeon selection among Iranian patients, assessing decision-making factors, trust perceptions, accuracy concerns, and demographic influences.

**Methods:**

A cross-sectional survey of 384 patients at maxillofacial surgery clinics in Isfahan, Iran (September–November 2023), was conducted using structured questionnaires to collect data on demographics, surgeon selection pathways, social media use, trust, and accuracy concerns. Descriptive statistics, *χ*^2^ tests, one-sample *t* tests, and multiple linear regression were conducted using SPSS Version 26 to analyze platform impact and predictive variables.

**Results:**

Personal recommendations dominated surgeon selection (239/384, 62.2%), significantly outweighing Google (75/384, 19.5%) and Instagram (11/384, 2.9%; *χ*²=214.3, *P*<.001). Google and Instagram were used by 160 (41.7%) and 119 (31.0%) patients, respectively; however, their decision-making impact was low with (mean scores: Google 2.27 (0.82), Instagram 2.14 (SD 0.79) on a 1‐5 scale; *t* tests: *P*<.001). Patient-generated content drove trust, with reviews valued by 144 (37.5%) for Google and 157 (40.9%) for Instagram, and testimonials by 174 (45.3%) for Instagram. Professional credentials influenced 116 (30.2%) participants for Google. Accuracy concerns were moderate; (means values of Google 2.84 (SD 0.91), Instagram 2.85 (SD) 0.88; *P*<.05). Regression identified recommendations (*β*=.42, *P*<.001), credential trust (*β*=.19, *P*=.002), and review authenticity (*β*=.14, *P*=.02) as predictors, while social media use was not a significant predictor (*P*=.32). Participants were predominantly female (233/384, 60.7%), aged 21‐30 years (117/384, 30.5%), employed (159/384, 41.4%), with moderate income (201/384, 52.3%), and no prior surgery (205/384, 53.4%). Instagram use was higher among younger patients (21‐30 years: 48/117, 41.0%; *χ*²=12.4, *P*=.006).

**Conclusions:**

Social media plays a supplementary role in the selection of maxillofacial surgeons in Iran, with traditional networks prevailing due to cultural trust and low health literacy (adequacy in 43% patients). The emphasis on credible reviews and credentials underscores the need for verified digital content. Contrasting with the digital reliance on aesthetic surgery, these findings advocate for verified profiles, patient education portals, and culturally tailored strategies to enhance trust and patient-centered care.

## Introduction

Social media has transformed health care decision–making by offering patients access to diverse information sources beyond traditional referrals. Platforms such as Google and Instagram play distinct yet complementary roles in shaping provider selection [1].

Google, as a search engine, aggregates patient reviews, professional credentials, and clinic websites, enabling patients to evaluate maxillofacial surgeons based on structured data such as ratings and qualifications [2].

Instagram, a visual platform, showcases before-and-after photos, patient testimonials, and surgeon branding, leveraging aesthetic appeal to influence decisions, particularly in specialties like maxillofacial surgery, where outcomes are both functional and cosmetic [3].

Globally, 59% of adults use online platforms for health decisions, with 43% relying on patient-generated content [4] . In maxillofacial surgery, where trust and expertise are critical, patients increasingly integrate these digital tools with personal recommendations, although the balance varies by cultural context [3].

The role of Google in health care is pivotal due to its accessibility and comprehensive search capabilities. Approximately 80% of health-related queries begin with Google, with patients seeking surgeon credentials, hospital affiliations, and peer reviews [5]. Google My Business profiles, featuring star ratings and patient feedback, significantly influence trust, with 70% of patients prioritizing high ratings [6]. However, concerns about review authenticity and algorithmic biases persist, as manipulated ratings can mislead patients [7].

Conversely, Instagram conversely, thrives on visual storytelling. In aesthetic surgery, 64% of patients were influenced by Instagram’s before-and-after imagery, a trend relevant to maxillofacial surgery [8]. Surgeons use Instagram to post educational content and case studies, achieving high engagement [9]. However, the emphasis of Instagram on aesthetics can oversimplify complex procedures, raising accuracy concerns [10].

In Iran, health care decisions are shaped by collectivist cultural norms, prioritizing familial and community referrals over digital sources. With 53% social media penetration compared to 80% in Western nations, and low health literacy [11], reliance on Google and Instagram is limited.

Iranian patients value verified credentials on Google and authentic testimonials on Instagram, though rural digital access constraints and urban service concentration hinder adoption [12].

Recent studies highlight these dynamics: Zhang et al [13] found that 68% of collectivist society patients prefer offline referrals, while Chegini et al [14] noted trust in peer-endorsed digital content [14].

Despite these insights, a critical research gap remains. No prior studies have quantitatively compared the influence of Google and Instagram versus personal recommendations on maxillofacial surgeon selection in Iran, particularly regarding trust, decision-making factors, and information accuracy. Existing literature focuses on telemedicine or primary care, overlooking specialty-specific dynamics in Iran’s culturally distinct health care context [12,15].

This study aims to address this gap by quantitatively assessing the relative influence of Google and Instagram versus personal recommendations on maxillofacial surgeon selection among Iranian patients. By analyzing trust perceptions, decision-making drivers, and accuracy concerns, it seeks to develop evidence-based, culturally tailored strategies to enhance digital trust and patient-centered care in Iran’s evolving health care system.

## Methods

This descriptive-analytical cross-sectional study was conducted in Isfahan, Iran, a metropolitan city with approximately 2.1 million residents and a center for advanced medical care. A cluster sampling approach was employed for representativeness.

Recruitment took place at three outpatient maxillofacial surgery clinics affiliated with the Faculty of Dentistry at Islamic Azad University, Isfahan, selected for their high patient volume (100‐150 weekly visits) and diverse patient demographics (socioeconomic and cultural backgrounds). Located in central Isfahan, these clinics offer functional procedures (eg, orthognathic surgery, fracture repair) and cosmetic interventions (eg, facial contouring, genioplasty), serving urban and rural patients.

Data collection took place from September 1 to November 30, 2023, during clinic hours (8:00 AM to 4:00 PM, Sunday to Thursday), using private consultation rooms for confidential survey administration with minimal disruption to operations.

The sample size was calculated based on a population exceeding one million, using a proportion estimate of 0.5, a margin of error of 0.05, and a 95% confidence level, resulting in 384 participants to ensure statistical power and generalizability. No major disruptions (eg, public health restrictions) affected clinic access. Eligible patients were adults aged 18 or older who had visited a maxillofacial surgeon for consultation or surgery and had used social media (eg, Google, Instagram) for health care choices within the past six months, with sufficient literacy to complete the questionnaire. Exclusions included patients under 18, those with cognitive or language barriers (eg, severe developmental disorders, non-Persian speakers without translation support), nonresidents of Iran, those who withdrew consent, provided incomplete responses, or did not use social media for surgeon selection.

Consecutive sampling was applied within selected clinics to reduce bias. Trained receptionists screened patients at check-in using a standardized checklist. Two research assistants approached eligible patients in waiting areas, providing a Persian-language information sheet detailing the study’s purpose, voluntary participation, confidentiality, and survey duration (10‐15 min).

### Data Collection Instrument

A structured, researcher-designed questionnaire with 25 items covered four domains:

1. Demographic Information: Age, gender, education, socioeconomic status.
2. Social Media Usage: Frequency of interaction with platforms like Google and Instagram
3. Factors Influencing Surgeon Selection: Impact of peer reviews, social media content, and feedback
4. Satisfaction and Influence: Satisfaction with the chosen surgeon and the role of digital information

Trust and accuracy scores were calculated as mean composite scores from four 5-point Likert-scale items (1=strongly disagree, 5=strongly agree) within the Satisfaction and Influence domain, assessing the perceived trustworthiness and reliability of social media information used for surgeon selection.

Ten experts confirmed the content’s validity (Content Validity Ratio [CVR]=0.8, Content Validity Index [CVI]=1.0). Reliability was assessed via test-retest with 40 patients two weeks apart (Cronbach α=0.704).

Questionnaires were completed in private clinics, with trained assistants ensuring honest responses. Data collection was monitored over three months for consistency and completeness.

### Reporting Guideline Adherence

This study adheres to the STROBE (Strengthening the Reporting of Observational studies in Epidemiology) checklist for cross-sectional studies, ensuring transparent reporting of study setting, eligibility criteria, recruitment methods, response rates, and potential biases [16], aligning with Q1 journal reporting standards.

### Statistical Analysis

Data were analyzed using descriptive and inferential methods. Demographic variables (eg, age, gender, education) were summarized using means (SD) and percentages. Normality was assessed via Shapiro-Wilk tests. Parametric (eg, two-tailed *t* tests, ANOVA) or nonparametric tests (eg, Mann-Whitney U) were applied based on data distribution. Associations between social media use and surgeon selection were evaluated using Spearman or Pearson correlations. Multiple linear regression models examined the influence of social media usage (eg, Google, Instagram) on decision-making, controlling for age and gender as confounders. Analyses were performed using SPSS software (version 26.0; IBM Corp), with *α*=.05.

### Ethical Considerations

The study protocol underwent ethical review and was approved by the Institutional Review Board (IRB) of Islamic Azad University, Isfahan, under the ethical approval code (IAU.YAZD.REC.1403.092). All 384 participants provided written informed consent before completing the survey.

During recruitment at maxillofacial surgery clinics, trained research assistants provided a Persian-language information sheet outlining the study’s purpose, procedures, voluntary participation, and right to withdraw without consequence. Consent forms were signed and collected before survey administration. As this study involved primary data collection, no secondary analysis was performed, rendering additional consent for such purposes inapplicable.

Participant privacy was safeguarded through anonymization of all survey responses at the point of collection. No personally identifiable information (eg, names, addresses, or contact details) was recorded. Surveys were assigned unique numeric codes for data entry. Paper-based surveys were stored in a locked cabinet, and digitized data were maintained on a secure, password-protected server accessible only to authorized researchers. After transcription, paper surveys were securely shredded to prevent data breaches.

No financial or material compensation was offered to participants, given the survey’s brief duration (10‐15 min) and its administration during routine clinic visits. This approach minimized the risk of coercion and aligned with ethical guidelines for low-burden, voluntary research participation.

The manuscript and supplementary materials contain no images of individual participants or users, ensuring no risk of identification. All reported data are aggregated (eg, percentages, means, and statistical summaries), eliminating the need for additional consent for visual content or submission of related consent forms.

## Results

This study analyzed responses from 384 patients visiting maxillofacial surgery clinics in Isfahan, Iran, during autumn 2023, to assess the influence of social media on surgeon selection.

### Demographic Characteristics and Surgical Experience

The mean age of participants was 29.8 (SD 8.7) years and median of 28 (IQR 18‐65) years, with a skew toward younger individuals. A higher proportion of participants were female (60.7%). Education was diverse, with most holding a high school diploma or higher (65.1% cumulative). Employment was balanced, with 41.4% employed, and income was moderate, with 45.3% earning 10‐20 million IRR monthly. Over half (53.4%) had no prior maxillofacial surgery.

### Pathways to Surgeon Selection and Social Media Use

Personal recommendations were the primary pathway to surgeon selection, with Google and Instagram playing supplementary roles. A discrepancy in “Pathways to Surgeon” frequencies (374/384 responses) was due to 10 missing responses from an optional question. Social media engagement was moderate, with Google being used more frequently than Instagram in surgeon searches (Table 1).

**Table 1.** Frequency and percentage distribution of pathways and social media use for selecting maxillofacial surgeons in a cross-sectional study of 384 patients in Isfahan, Iran, September–November 2023.

Category and subcategories	Participants (N=384) , n (%)
Pathway to surgeon	
Friend or acquaintance recommendation	239 (62.2)
Google Search	75 (19.5)
Instagram	11 (2.9)
Other social media	11 (2.9)
Other	48 (12.5)
Missing responses	
Not applicable	10 (2.6)
Google use for search	
Yes	160 (41.7)
No	224 (58.3)
Instagram use for search	
Yes	119 (31.0)
No	265 (69.0)

### Decision-Making and Trust Factors on Social Media

Google users prioritized user reviews and official credentials, while Instagram users valued patient testimonials and real patient experiences (Table 2). *χ*^2^ tests showed significant differences between platforms in decision-making, selection, and trust factors (all *P*<.001), indicating distinct user behaviors.

**Table 2.** Comparison of decision-making, selection, and trust factors influencing maxillofacial surgeon selection on Google and Instagram in a cross-sectional study of 384 patients in Isfahan, Iran, September–November 2023, with *χ*^2^ test results.

Category and subcategories	Participants (Google), (n, %)	Participants (Instagram), (n, %)	*χ*^2^ (*df*)	*P* value
Initial decision-making			117.6 (4)	<.001
User reviews/patient testimonials	135 (35.2)	174 (45.3)		
Surgeon ranking/before-and-after images	85 (22.1)	78 (20.3)		
Website info/Surgeon engagement	32 (8.3)	22 (5.7)		
Photos-videos/educational videos	31 (8.1)	8 (2.1)		
Other	101 (26.3)	102 (26.6)		
Primary selection			76.4 (3)	<.001
Website Info/before-and-after images	30 (7.8)	44 (11.5)		
Rankings/positive reviews	105 (27.3)	157 (40.9)		
Positive reviews/Surgeon interactions	144 (37.5)	56 (14.6)		
Other	105 (27.3)	127 (33.1)		
Trust-building			92.3 (3)	<.001
Authentic Reviews / Patient Experiences	102 (26.6)	153 (39.8)		
Credentials / Procedure Videos	116 (30.2)	78 (20.3)		
Validated Info / Follower Count	53 (13.8)	27 (7.0)		
Other	113 (29.4)	126 (32.8)		

### Statistical Analysis of Influence and Concerns

The impact of Google and Instagram on decision-making, measured as mean composite scores from three 1‐5 Likert items per construct (eg, influence: perceived influence, usefulness, relevance; accuracy: reliability, trust), was low (both *P*s<.001), suggesting limited influence. Moderate concerns about information accuracy were noted (both *Ps*<.05) (Table 3). The large sample size supported parametric tests despite non-normal distributions.

**Table 3.** One-Sample *t* test results for impact of information and concerns about accuracy of Google and Instagram in influencing maxillofacial surgeon selection in a cross-sectional study of 384patients in Isfahan, Iran, September–November 2023.

Categories and variables	Participants (N=384), mean (SD)	*t* test (*df*)	*P* value
Impact of information			
Google	2.27 (1.22)	−11.808 (383)	<.001
Instagram	2.14 (1.20)	−14.022 (383)	<.001
Concerns about accuracy			
Google	2.84 (1.18)	−2.628 (383)	.009
Instagram	2.85 (1.26)	−2.315 (383)	.02

### Multiple Linear Regression Analysis

Multiple regression analysis (Table 4) revealed that personal recommendations and trust in official credentials significantly predicted decision-making outcomes (*P*<.001and *P*=.002, respectively), while Google and Instagram use did not significantly predict outcomes (*P*>.05). The model fit was acceptable (F_5, 378_=14.32, *P*<.001, *R*²=0.16).

**Table 4.** Multiple linear regression analysis of factors predicting decision-making outcomes for maxillofacial surgeon selection in a cross-sectional study of 384patients in Isfahan, Iran, September–November 2023, controlling for age and education.

Predictor	β	*t* test	*P* value
Personal recommendations	.42	7.98	<.001
Google use	.09	1.58	.12
Instagram use	.06	1.18	.24
Trust in credentials	.19	3.09	.002
Age	−.03	−0.59	.56
Education	0.04	0.82	.41

## Discussion

### Principal Results

This study investigated the influence of social media on maxillofacial surgeon selection among 384 patients in Isfahan, Iran, during autumn 2023. The main findings reveal that personal recommendations significantly dominated surgeon selection, with 62.2% of patients relying on friends or acquaintances, compared to 19.5% using Google and only 2.9% using Instagram. While Google (41.7%) and Instagram (31.0%) were used as supplementary tools, their impact on decision-making was significantly below average, as evidenced by mean scores of 2.27 and 2.14, respectively, on a 1‐5 scale. Patient-generated content, such as reviews and testimonials, alongside professional credentials, emerged as critical factors in decision-making and trust-building across both platforms. However, moderate concerns about information accuracy (means of 2.84 for Google and 2.85 for Instagram, respectively) suggest skepticism toward digital sources, reinforcing the primacy of traditional networks.

### Comparison With Prior Work

The prominence of personal recommendations aligns with prior research emphasizing interpersonal trust in health care decisions. The preference for traditional networks in Iran likely stems from cultural factors, such as collectivist values prioritizing familial and community ties, and high social trust in personal referrals over impersonal digital sources. Low health literacy, prevalent in Iran, with only 43% of adults demonstrating adequate health knowledge [17] may further limit reliance on online information, as patients defer to trusted acquaintances for guidance. A recent study by Wang et al [18] found that 68% of patients in collectivist societies preferred offline referrals, mirroring our 62.2% reliance on personal networks, compared to 20% using online platforms. Similarly, Bhatt et al [19] reported that 55% of Asian patients favored word-of-mouth over digital reviews, consistent with our findings.

The supplementary role of social media resonates with global trends. Xiong et al [20] noted that 43% of Chinese patients used online searches; however, offline sources guided final decisions, paralleling our Google (41.7%) and Instagram (31.0%) usage. Zheng et al [21] showed that only 28% of patients globally trust online reviews for surgical choices , aligning with our limited social media impact (*P*<.001), reinforcing the secondary role of digital platforms. The influence of patient-generated content’s (eg, 37.5% for Google reviews, 45.3% for Instagram testimonials) mirrors findings by Thoms et al [22], where 50% of patients valued authentic reviews, though trust was tempered by authenticity concerns, reflected in our moderate accuracy scores (approximately 2.85). This skepticism aligns with Chow et al [23], who reported that 40% of patients questioned digital health information due to a lack of verification.

In contrast, Instagram’s minimal role (2.9%) diverges from aesthetic surgery contexts. ElAbd [8] et al found that 64% of plastic surgery patients were influenced by Instagram’s visual content, likely due to cosmetic surgery’s emphasis on aesthetics unlike the functional focus of maxillofacial procedures in our study. Cultural attitudes in Iran, where social media penetration is lower (53% vs 80% in Western nations) [24], may further reduce Instagram’s impact, reinforcing traditional networks.

The role of professional credentials (30.2% for Google) corroborates the findings of Daraz et al [25], where 48% prioritized verified qualifications online. Moderate accuracy concerns highlight a trust gap, consistent with findings by Sorensen et al [26], who noted patients’ demand for verified digital content to bridge credibility issues.

### Implications for Clinical Practice

The dominance of personal recommendations underscores the need for maxillofacial surgeons to maintain robust referral networks with colleagues and satisfied patients. To enhance their online presence, surgeons should prioritize authentic patient testimonials, verified credentials, and transparent procedure information on platforms like Google and Instagram. For example, creating verified Google Business Profiles with certified reviews or Instagram posts showcasing patient outcomes and qualifications can build trust. Hospitals and clinics could develop patient education portals with validated content to address accuracy concerns, particularly for populations with low health literacy. Professional organizations should advocate for standardized online verification processes, such as digital badges for board-certified surgeons, to counter skepticism. These strategies can complement traditional networks, leveraging digital tools to reach tech-savvy patients while maintaining credibility.

### Limitations

The study’s focus on Isfahan limits generalizability, as rural or regional variations in Iran may differ. The cross-sectional study design prevents causal inferences about digital influence over time. Additionally, the participants’ moderate income and education may not reflect less affluent or less educated groups, potentially underestimating digital access barriers.

### Future Directions

Longitudinal studies should track social media’s evolving role in Iran as digital literacy improves. Comparative research across surgical specialties and cultural contexts could clarify drivers of platform use. Exploring interventions such as blockchain-verified reviews or artificial intelligence–driven content validation may address accuracy concerns, enhancing digital tools’ role in surgeon selection.

### Conclusion

In conclusion, personal recommendations remain the cornerstone of maxillofacial surgeon selection in Isfahan, with social media playing a limited, supplementary role shaped by patient reviews and credentials. These findings, consistent with global patterns, yet distinct in their low Instagram reliance, highlight the enduring trust in traditional networks and the need for credible digital enhancements in health care decision-making.

## References

[1] Graham AL, Cobb CO, Cobb NK Handbook of Health Decision Science.

[2] Lali MIU, Mustafa RU, Saleem K, Nawaz MS, Zia T, Shahzad B (2017). Finding healthcare issues with search engine queries and social network data. Int J Semant Web Inf Syst.

[3] Nguyen MN, Yeung JG, Randall Y, Boesze-Battaglia K, Panchal N (2021). Oral and maxillofacial surgery social media boom: potential concerns of social media use for the surgeon. J Oral Maxillofac Surg.

[4] Lim MSC, Molenaar A, Brennan L, Reid M, McCaffrey T (2022). Young adults’ use of different social media platforms for health information: insights from web-based conversations. J Med Internet Res.

[5] Kobrinskii BA Search engine decision-relevant information and exchange with the information system.

[6] Chen TT, Wu CI, Chiu MHP (2022). Factors associated with the patient/client use of report cards, physician rating websites, social media, and google for hospital and physician selection: a nationwide survey. Healthcare (Basel).

[7] Van Haasteren A (2019). Trust in digital health [Thesis].

[8] ElAbd R, Alghanim K, Alnesef M, Alyouha S, Samargandi OA (2023). Aesthetic surgery before-and-after photography bias on Instagram. Aesthetic Plast Surg.

[9] Ma B, Rojas EM, Li AYZ, Kinard BE (2024). To what extent is oral and maxillofacial surgery educational content posted on Instagram?. Int J Oral Maxillofac Surg.

[10] Rekawek P, Wu B, Hanna T (2021). Minimally invasive cosmetic procedures, social media, and oral-maxillofacial surgery: use of trends for the modern practice. J Oral Maxillofac Surg.

[11] Haeri-Mehrizi A, Mohammadi S, Rafifar S (2024). Health literacy and mental health: a national cross-sectional inquiry. Sci Rep.

[12] Rezaei N, Moghaddam SS, Farzadfar F, Larijani B (2023). Social determinants of health inequity in Iran: a narrative review. J Diabetes Metab Disord.

[13] Zhang S, Zhou H, Zhu Y (2024). Have we found a solution for health misinformation? A ten-year systematic review of health misinformation literature 2013-2022. Int J Med Inform.

[14] Chegini Z, Kakemam E, Behforoz A, Lotfollah-Zadeh F, Jafari-Koshki T, Khodayari Zarnag R (2022). Impact of patient communication preferences on the patient trust in physicians: a cross-sectional study in Iranian outpatient’s clinics. J Patient Exp.

[15] Ashtarian K, Etemadi M (2023). Popular diffusion as an instrument for overcoming barriers to digital health in Iran: the critical role of the pandemic. IJHG.

[16] Skrivankova VW, Richmond RC, Woolf BAR (2021). Strengthening the Reporting of Observational Studies in Epidemiology Using Mendelian Randomization: The STROBE-MR Statement. JAMA.

[17] Haghdoost AA, Karamouzian M, Jamshidi E (2019). Health literacy among Iranian adults: findings from a nationwide population-based survey in 2015. East Mediterr Health J.

[18] Wang R, Huang Y, Zhang X, Yao Y (2023). Online dialogue with medical professionals: an empirical study of an online “Ask the Doctor” platform. Int J Med Inform.

[19] Bhatt NR, Czarniecki SW, Borgmann H (2021). A systematic review of the use of social media for dissemination of clinical practice guidelines. Eur Urol Focus.

[20] Xiong Z, Zhang L, Li Z, Xu W, Zhang Y, Ye T (2021). Frequency of online health information seeking and types of information sought among the general Chinese population: cross-sectional study. J Med Internet Res.

[21] Zheng B, Cai X (2025). Fundamentals of digital surgery: surgeon-centered data enchantment for presurgical planning, intraoperative performance and decision making. Laparosc Endosc Robot Surg.

[22] Thoms B, Botts N, Eryilmaz E Examining trust and consent models for patient-generated health data-sharing and incentives.

[23] Chow E, Virani A, Pinkney S (2024). Caregiver and youth characteristics that influence trust in digital health platforms in pediatric care: mixed methods study. J Med Internet Res.

[24] Ahamed A, Gong W (2022). What drives high penetration rates of social media a qualitative comparative analysis across countries. JBM.

[25] Daraz L, Morrow AS, Ponce OJ (2019). Can Patients trust online health information? A meta-narrative systematic review addressing the quality of health information on the internet. J Gen Intern Med.

[26] Sørensen K (2024). Fostering digital health literacy to enhance trust and improve health outcomes. Computer Methods and Programs in Biomedicine Update.

